# Functional role of post-translational modifications of Sp1 in tumorigenesis

**DOI:** 10.1186/1423-0127-19-94

**Published:** 2012-11-14

**Authors:** Wen-Chang Chang, Jan-Jong Hung

**Affiliations:** 1 Institute of Basic Medical Sciences, College of Medicine, National Cheng-Kung University, Tainan 701, Taiwan; 2 Department of Pharmacology, College of Medicine, National Cheng-Kung University, Tainan 701, Taiwan; 3 Institute of Bioinformatics and Biosignal Transduction, College of Bioscience and Biotechnology, National Cheng-Kung University, Tainan 701, Taiwan; 4 Center for Infection Diseases and Signal Transduction Research, National Cheng-Kung University, Tainan, 701, Taiwan; 5 Graduate Institute of Medical Sciences, College of Medicine, Taipei Medical University, Taipei, Taiwan

## Abstract

Specific protein 1 (Sp1), the first transcription factor to be isolated, regulates the expression of numerous genes involved in cell proliferation, apoptosis, and differentiation. Recent studies found that an increase in Sp1 transcriptional activity is associated with the tumorigenesis. Moreover, post-translational modifications of Sp1, including glycosylation, phosphorylation, acetylation, sumoylation, ubiquitination, and methylation, regulate Sp1 transcriptional activity and modulate target gene expression by affecting its DNA binding activity, transactivation activity, or protein level. In addition, recent studies have investigated several compounds with anti-cancer activity that could inhibit Sp1 transcriptional activity. In this review, we describe the effect of various post-translational modifications on Sp1 transcriptional activity and discuss compounds that inhibit the activity of Sp1.

## Introduction

The transcription factor Sp1 (specificity protein 1) belongs to the family of Sp/KLF (Krüppel-like factor) transcription factors. It is the first transcription factor purified and cloned from mammalian cells [1]. The human Sp1 gene maps to 12q13.1 and encodes a protein of 785 amino acids. The protein contains an N-terminal transactivation domain, which recruits the basal transcriptional machinery complex to the target promoter, and a C-terminal DNA binding domain, which contains three Cys_2_His_2_-type zinc finger DNA binding motifs required for recognizing GC-rich (GGGGCGGGG) promoter sequences [2]. Sp1 regulates thousands of genes, such as those encoding vascular endothelial growth factor (VEGF), p21^CIP1/WAF1^, 12(*S*)-lipoxygenase, phosphatase 2A (PP2A), and Sp1 itself. Thus, Sp1 is important for a variety of physiological processes, including angiogenesis, cell cycle progression, inflammation, and senescence [3-6]. Dysregulation of Sp1 is observed in many cancers and neurodegenerative disorders. Sp1 gene knockout is embryonic lethal at the 11^th^ day of gestation [7].

Sp1 has been reported to affect the tumorigenesis of many cancer types by modulating the expression of its target genes, which include oncogenes and tumor suppressor genes. A recent study demonstrated that both single nucleotide polymorphism (SNP) 285 and SNP 309 in MDM2 (murine double mutant 2) are found in breast cancer and can affect the binding of Sp1 to the MDM2 promoter [8]. In addition, the insulin-like growth factor (IGF) system plays an important role in the biology of breast cancer. A previous study revealed that caveolin-1 up-regulates IGF-1 receptor gene transcription in breast cancer cells via Sp1- and p53-dependent pathways [9]. A number of E2-responsive genes involved in nucleotide biosynthesis and cell cycle progression are dependent on estrogen receptor (ER)-α/Sp1 interactions [10]. Moreover, it was reported that phytoestrogen regulation of the vitamin D3 receptor promoter is regulated by Sp1. Finally, in the presence of the estrogen receptor antagonist ICI 182780, ER-α and histone deacetylases (HDACs) dissociate from Sp1, resulting in increased histone acetylation and the induction of p21 expression [11,12]. In lung cancer, we recently found that Sp1 accumulates in the early stage and then declines in the late stage, which is important for lung cancer cell proliferation and metastasis. Sp1 was required for lung tumor growth but it suppressed metastasis by inducing E-cadherin expression. The clinical implication of these results is that Sp1 inhibition is seemingly inappropriate for all patients with lung cancer ranging from stage I to IV [13]. In addition, the deregulation of DNA (cytosine-5-)-methyltransferase 1 (DNMT1) is associated with a gain in the transcriptional activation of Sp1 and a loss in the repression of p53. DNMT1 overexpression might result in the epigenetic alteration of multiple tumor suppressor genes and ultimately lead to lung tumorigenesis and poor prognosis [14]. The cytosolic phospholipases A2 (cPLA2) expression is regulated by Sp1 and c-Jun in lung cancer cells [15]. In colon cancer, HDAC inhibitors can induce cancer cell apoptosis by activating Krüppel-like factor 4, and Sp1/Sp3 can increase the apoptotic sensitivity of colon cancers to histone deacetylase inhibitors [16]. In addition to the cancer types described here, Sp1 is implicated in other cancer types and in tumorigenesis by mediating the expression of many oncogenes [17-19].

### Effects of post-translational modifications of Sp1 on its transcriptional activity

**Figure 1:**
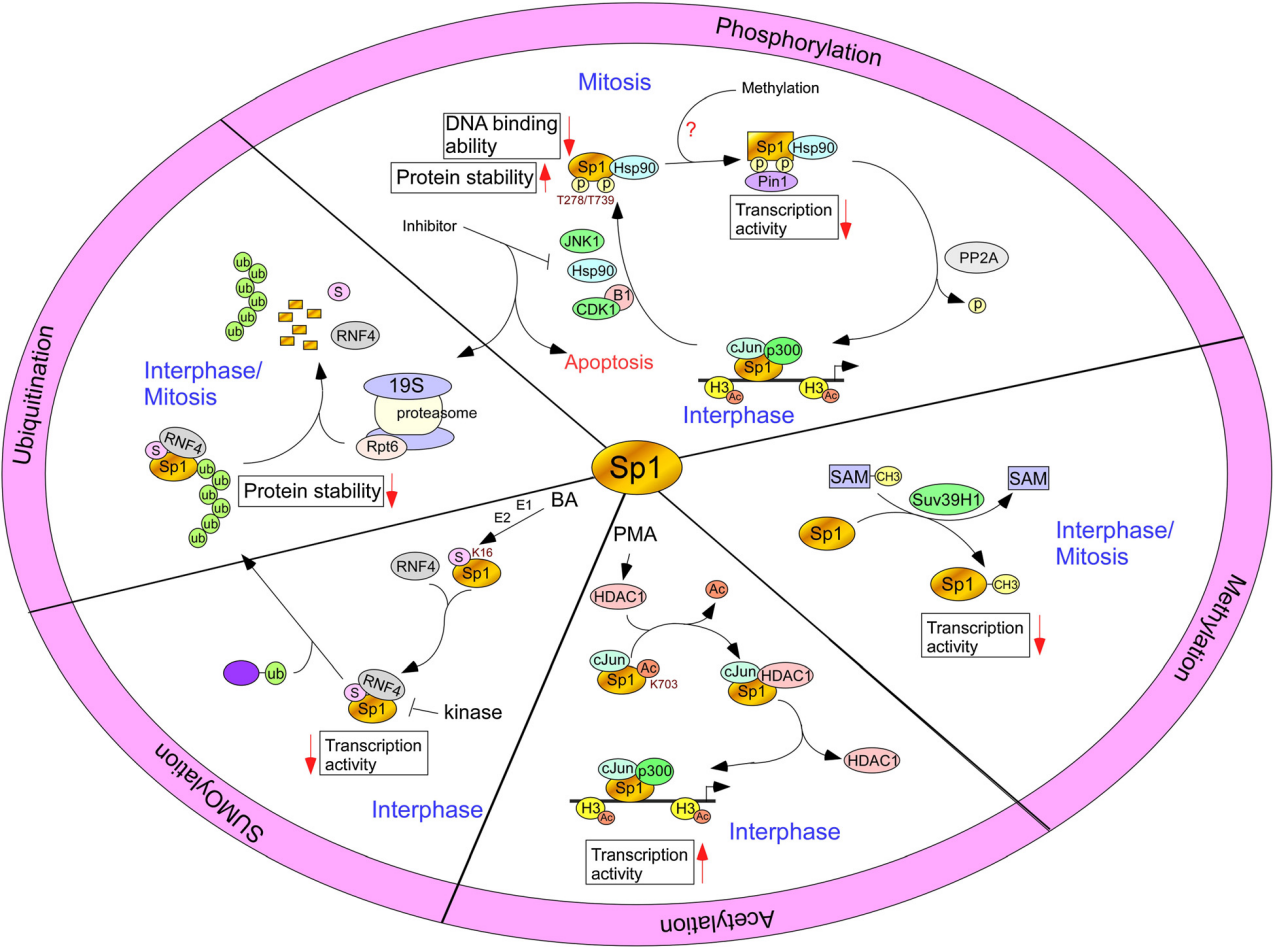
The schematic diagram illustrating that various modifications in Sp1 modulate its transcriptional activity.

The involvement of Sp1 in the development of various cancer types is well known. The transcriptional activity of Sp1 is modulated by post-translational modifications that regulate Sp1 protein level, transactivation activity, and DNA binding affinity [20]. Here, we describe the Sp1 modifications and their effects on Sp1 transcriptional activity as indicated in Figure 1.

### Phosphorylation

There are 23 putative phosphorylation sites (from PhosphoSitePlus®) among the 785 amino acids of Sp1. Many of these sites have been shown to positively or negatively affect Sp1 transcriptional activity by modulating Sp1’s DNA binding affinity, transactivation activity, or total protein level in a manner dependent on the position of the residue or the condition of the cell [20].

### Effect on Sp1 DNA binding activity

Sp1 is phosphorylated at Ser59 by cyclin A-cyclin dependent kinase (CDK). In in vitro and in vivo phosphorylation studies, cyclin A-CDK complexes can phosphorylate Sp1, and the phosphorylation site is located within the N-terminal region of the protein [21]. The DNA binding activity of Sp1 is elevated in cells overexpressing cyclin A [21]. How a phosphorylation site located in the N-terminus of Sp1 affects the zinc finger domains in the C-terminus of Sp1 is still unknown and needs further studies. We recently reported that Sp1 is a mitotic substrate of CDK1/cyclin B1 and is phosphorylated by CDK1/cyclin B1at Thr739. Phosphorylation of Sp1 reduces its DNA-binding ability and facilitates the chromatin condensation process during mitosis [22]. At the end of mitosis and the beginning of interphase, Sp1 is dephosphorylated by PP2A and returned to the chromatin. These results indicate that cancer cells use CDK1 and PP2A to regulate the movement of Sp1 in and out of the chromosomes during cell cycle progression. Sp1 is also phosphorylated at Thr668 by casein kinase II (CKII) to decrease its DNA binding activity. In addition, treatment of K562 cells with okadaic acid increases Sp1 phosphorylation and inhibits its DNA binding activity, suggesting that steady state levels of Sp1 phosphorylation are established by a balance between kinase and phosphatase activities [23]. However, phosphorylation of Sp1 at Thr668, Ser670, and Thr681 by protein kinase C zeta (PKC-ζ) is required for Sp1-dependent platelet-derived growth factor-D activation in response to angiotensin II [24]. Further studies are needed to explain why the phosphorylation of Sp1 in this region has different effects on Sp1 transcriptional activity.

### Effect on Sp1 transactivation activity

Many of the phosphorylation sites shown in Table 1 could regulate the transcriptional activity of Sp1 by altering its interaction with other proteins, such as p300 and HDACs, which can positively or negatively regulates the Sp1 target genes.

**Table 1:** Post-translational modification of Sp1

**PTM**	**Modified sites**	**Modified enzymes**	**Functions**	**Ref.**
**Phosphorylation**	Ser59	CDK2	Increase Sp1 DNA binding activity	[21]
	Ser101	ATM	Involve in DNA damage and repair	[25]
	Ser131	DNA-PK	Upregulate expression of the HIV-1 Tat protein.	[26]
	Thr266	Ras-MAPK	Increase expression of apolipoprotein A-I	[27]
	Thr278/739	JNK1	Increase Sp1 protein stability	[28]
	Thr453/739	P42/p44 MAPK	Enhance VEGF expression	[29]
		ERK1/2	Repress PDGFR-α transcription	[30]
		ERK1/2	Inhibit RECK expression and promote cell invasion	[31]
	Ser641	PI3K/PKCζ	Increase luteinizing hormone receptor (LHR) transcription	[32]
	Thr668	CKII	Decreased Sp1 DNA binding acticity	[23]
	Thr668/Ser670/Thr681	PKCζ	Increase PDGF transcription	[24]
	Thr739	CDK1	Decrease Sp1 binding activity	[22]
**Sumoylation**	Lys16	Sumo-1	Enhance Sp1 degradation	[33,34]
**Methylation**	ND	ND	Recruit Suv39H1 and HDAC1 to induce chromatin remodeling	[35]
**Acetylation**	Lys703	P300	Recruit HDAC1 and p300 to the promoter of the 12(S)-lipoxygenase	[36]
**Glycosylation**	Leu56/57	ND	Sp1 degradation	[37]
	Ser612/641/698/702, Thr640	ND	Regulate transcriptional activity of Sp1	[38]

ND: non-determine.

### Effect on Sp1 protein stability

Recent studies have shown several phosphorylation sites related to Sp1 protein stability. Sp1 is highly phosphorylated during mitosis. Phosphorylation of Sp1 by c-Jun N-terminal kinase 1 (JNK1) at Thr278/739 is important for the maintenance of Sp1 in daughter cells. JNK1 is also highly activated during tumorigenesis and correlates with Sp1 protein level. These results indicate that JNK1 activation is necessary to phosphorylate Sp1 and to shield Sp1 from the ubiquitin-dependent degradation pathway during mitosis in tumor cell lines [28]. In addition, heat shock protein 90 (HSP90) can interact with Sp1 during mitosis to alter the phosphorylation of Sp1 at Thr278/739 by JNK1 and increase Sp1 protein stability [39]. These results indicate that Hsp90 might interact with JNK1 and stabilize it during mitosis, and thereby enhance the phosphorylation of Sp1. Our recent studies indicate that phosphorylation of Sp1 at Thr739 increases its stability by preventing interaction with RING finger protein 4 (RNF4), thus shielding Sp1 from proteasome-dependent degradation [33]. However, the manner in which phosphorylated Sp1 is shielded from interaction with RNF4 needs to be clarified.

In addition, several phosphorylation sites of Sp1 have been probed, and their roles have been elucidated, but the detailed mechanism is still unknown. It was reported that Sp1 can be phosphorylated by ataxia telangiectasia mutated (ATM) at Ser101 under DNA damage conditions [25]. Sp1 with a Ser101Ala mutation is not significantly phosphorylated in response to damage, and it can not restore sensitivity to DNA damage [25]. These results indicate that Sp1 is an ATM substrate that plays a role in the cellular response to DNA damage. Ser101 phosphorylation did not affect transcriptional activity from the Sp1 responsive promoter, but it did promote co-localization of Sp1 with ATM phosphorylated at Ser1981. These observations suggest that Sp1 phosphorylated at Ser101 might play a role in DNA repair at damage sites rather than functioning in transcriptional regulation, but further evidence is required to substantiate this possibility. The mechanism by which Sp1, phosphorylated at Ser101 by ATM, affects DNA damage needs to be elucidated. In addition, it was reported that EGF stimulation of apoA-I expression is mediated solely by the Ras-MAP kinase cascade and that enhanced activity of this pathway requires Sp1 with an intact phosphorylation site at Thr266 [27]. It was also shown that trichostatin A (TSA) causes marked phosphorylation of Sp1 at Ser 641 by PKCζ in JAR and MCF-7 cells, indicating that phosphorylation of Sp1 by phosphatidylinositol 3-kinase (PI3K)/PKCζ is critical for TSA-activated lutropin/choriogonadotropin receptor (LHR) gene expression [32]. It was also reported that p42/p44 mitogen-activated protein kinase (MAPK) directly phosphorylates Sp1 at Thr453 and Thr739 in vitro and in vivo, and mutation of these sites to alanine decreases Sp1 transcriptional activity [29]. In addition, fibroblast growth factor 2 (FGF-2) stimulates Sp1 phosphorylation in an extracellular signal-regulated kinase (ERK) 1/2- but not p38-dependent manner, and enhances Sp1 recruitment to the platelet-derived growth factor receptor alpha (PDGFR-α) promoter region [30]. It was also found that Sp1, phosphorylated at Thr453 and Thr739 by ERK, binds preferentially to the reversion-inducing cysteine-rich protein with Kazal motifs (RECK) promoter, and then recruits HDAC1 to silence RECK gene expression [31]. According to these studies, phosphorylation at Thr453 and Thr739 by ERK1/2 in interphase might modulate the transactivation activity of Sp1. Taken together, the results suggest that phosphorylation of Sp1 at Thr739 is very important. In interphase cells, it affects Sp1 transcriptional activity by modulating different regulatory factors, such as HDAC1. In mitotic cells, Thr739 phosphorylation controls the movement of Sp1 in and out of chromosomes to finish chromosome packaging by decreasing Sp1 DNA binding affinity. Finally, it stabilizes Sp1 by shielding it from interaction with RNF4.

### Sumoylation and ubiquitination

Sumoylation is a post-translational modification involved in various cellular processes such as nuclear-cytosolic transport, transcriptional regulation, protein stability, DNA repair, and apoptosis [40-44]. Sp1 can be sumoylated by SUMO-1 at Lys16. Sumoylation of Sp1 decreases its protein stability by altering its subcellular localization and recruiting regulatory particle triphosphatase 6 (RPT6) to increase ubiquitination. The sumoylation level of Sp1 is decreased in tumorous cervical tissues. These results suggest that Sp1 accumulation correlates with the inhibition of sumoylation during tumorigenesis [34]. In our recent study, we found that RNF4 acts as the E3 ubiquitin ligase and triggeres Sp1 sumoylation and ubiquitin-mediated proteolysis [33].

### Acetylation

Acetylation of histones, especially histone H3 and H4, is important for gene regulation [45]. Recently, several important non-histone proteins, such as p53 and Sp1, were reported to be acetylated [36,46,47]. We found that phorbol 12-myristate 13-acetate (PMA) induces the deacetylation of Sp1, and then increases Sp1 transcriptional activity at a target gene, 12(*S*)-lipoxygenase, by enhancing the recruitment of p300 to the promoter of 12(*S*)-lipoxygenase [36].

### Methylation

Methylation, a common histone modification, negatively affects gene expression by recruiting suppressor factors such as HDACs or DNA methyltransferases [48]. Recently, the functional activity of several important transcription factors was found to be modified by methylation [49,50]. We recently found that methylated Sp1 recruits Suv39H1 and HDAC1 to the promoter of Sp1 target genes to repress gene expression [35]. However, the enzyme that provides the methyltransferase activity and the residue(s) that is methylated are still unknown.

### Glycosylation

Many important transcription factors, including Sp1, a key promoter required for an optimal HSP response following stress, are glycosylated by the *O*-GlcNAc pathway [51]. Previous studies indicated that heat shock factor-1, HSP70, and HSP27 expression all rely on Sp1 binding to their promoter regions [52-54]. Exposure of either cell line to high-dose glutamine is sufficient to induce Sp1 glycosylation and regulation of its target genes, such as argininosuccinate synthetase [55]. However, under glucose starvation, stimulation with cyclic AMP (cAMP) results in nearly deglycosylation of Sp1, and Sp1 is rapidly proteolytically degraded by an enzyme that can be inhibited by specific proteasome inhibitors, lactacystin and *N*-acetyl-l-leucyl-l-leucyl-l-norleucinal (LLnL) [56]. It was reported that the chloride channel-2 (ClC-2) is induced by highly phosphorylated and highly glycosylated Sp1 [57]. In addition, high glucose (HG) concentrations can potentially stimulate the expression of genes associated with the development of diabetic nephropathy [58]. A recent report also indicated that insulin dynamically regulates calmodulin gene expression by sequential *O*-glycosylation and phosphorylation of Sp1 in liver cells [59]. In addition, hyperglycemia-induced mitochondrial superoxide overproduction increases hexosamine synthesis and *O*-glycosylation of Sp1, which activates the expression of genes that contribute to the pathogenesis of diabetic complications [60]. According to previous studies, there is a good correlation between glycosylation and phosphorylation of Sp1. Cross-talk between phosphorylation and glycosylation requires further investigation.

### Compounds inhibiting Sp1 transcriptional activity

Sp1 accumulates in most of cancer types and participates in their tumorigenesis. Several compounds with anti-tumor effects act by inhibiting Sp1 transcriptional activity as indicated in Table 2. A previous study found that *Oldenlandia diffusa* (OD) extracts strongly inhibit anchorage-dependent and -independent cell growth and induced apoptosis in ERα-positive breast cancer cells, and increase p53 expression as a result of enhanced binding of the ERα/Sp1 complex to the p53 promoter region [61]. Arsenic trioxide downregulates Sp1 expression and Sp-dependent gene expression in bladder cancer [62]. Celecoxib treatment reduces both Sp1 DNA binding affinity and its transactivating activity in pancreatic cancer [63]. Bortezomib decreases Sp1 protein levels, disrupts the interaction of Sp1 with NF-kappaB, and prevents binding of the Sp1/NF-kappaB complex to the DNMT1 gene promoter for repression in acute myeloid leukemia [64]. Curcumin was reported to induce proteasome-dependent degradation of Sp protein in bladder cancer [65]. GT-094 could downregulate Sp expression, and then repress Sp-regulated genes in colon cancer [66]. Treatment with 3,3^′^-diindolylmethane (DIM) could induce Sp1-mediated p21 expression in breast cancer cells [67]. Indole-3-carbinol (I3C) inhibits Sp1-mediated CDK6 expression in breast cancer [68]. Trichostatin A (TSA) treatment could increase Sp1-mediated IGF binding protein 3 (IGFBP-3) promoter activity in hepatoma [69]. Tolfenamic acid treatment induces the degradation of Sp1, Sp3, and Sp4 in pancreatic cancer [70]. Mithramycin A, the G-C specific DNA-binding drug, could bind to both consensus sequences and then prevent subsequent Sp1 binding to repress Sp1-mediated target gene expression [71]. Betulinic acid (BA) was reported to induce Sp1 degradation in prostate cancer [72]. Our recent study also indicates that BA could induce the sumoylation of Sp1 and then recruit the E3 ubiquitin ligase, RNF4, which contains SUMO-interacting motifs, to increase Sp1 ubiquitination, leading to Sp1 degradation in lung cancer [73].

**Table 2:** Compounds affected Sp1

**Drugs**	**Effect on Sp1**	**Cancer type**	**Ref.**
Oldenlandia Diffusa (OD)	Positive	Breast cancer	[61]
Arsenic trioxide	Negative	Bladder cancer	[62]
Celecoxib	Negative	Pancreatic cancer	[63]
Bortezomib	Negative	Acute myeloid leukemia	[64]
Curcumin	Negative	Bladder cancer	[65]
GT-094	Negative	Colon cancer	[66]
3,3^′^-diindolylmethane (DIM)	Positive	Breast cancer	[67]
Indole-3-carbinol (I3C)	Negative	Breast cancer	[68]
Trichostatin A (TSA)	Positive	Hepatoma	[69]
Tolfenamic Acid	Negative	Pancreatic cancer	[70]
Mithramycin A	Negative	ND	[71]
Betulinic acid	Negative	Prostate and lung cancer	[72,73]

ND: non-determine.

### Perspective

Although many studies have addressed the importance of Sp1 in tumorigenesis, much about Sp1 is still unknown. First, many post-translational modifications have been identified, but the cross-talk between these modifications needs further studies. Second, although many functions have been described, the relationship between Sp1 function and structure remains unknown. This may be due to instability of the Sp1 protein. Finally, a growing number of compounds inhibit Sp1 and suppress tumor formation, but a detailed mechanism and the side effects require further clarification.
